# Molecular Mechanisms of 6PPD and 6PPD-Q Toxicity in Neurodegenerative Diseases: A Network Toxicology and Experimental Validation Study

**DOI:** 10.3390/toxics14060504

**Published:** 2026-06-10

**Authors:** Ze Li, Yuyang Luo, Siyi Wang, Dingming Xue, Yixuan Zhang

**Affiliations:** 1 Department of Physiology, School of Basic Medical Science, Central South University, Changsha 410011, China; 2 Xiangya School of Medicine, Central South University, Changsha 410011, China; 3 Nanjing Institute of Environmental Sciences, Ministry of Ecology and Environment, Nanjing 210042, China

**Keywords:** 6PPD, 6PPD-quinone, environmental factor, neurodegenerative diseases, network toxicology, molecular docking

## Abstract

6PPD is a widely used tire antioxidant that readily transforms into its more toxic ozonation product, 6PPD-quinone (6PPD-Q). Both compounds are emerging environmental contaminants with potential neurotoxic risks, yet their molecular mechanisms in Alzheimer’s disease (AD) and Parkinson’s disease (PD) remain unclear. This study integrated network toxicology, molecular docking, transcriptomic validation, and experimental models to investigate their neurotoxic effects. In silico analyses predicted significant neurotoxicity and blood–brain barrier permeability for both compounds. Target prediction and PPI network analysis identified 145/121 overlapping targets with AD/PD for 6PPD and 120/100 for 6PPD-Q. Functional enrichment analysis suggested that 6PPD-associated targets were mainly enriched in axon regeneration-, p75NTR-, and AGE-RAGE-related pathways, whereas 6PPD-Q-associated targets were enriched in MAPK cascade-, endosomal TLR signaling-, and amyloid-β formation-related pathways. Molecular docking suggested favorable binding affinities between these compounds and several core targets, including MAP2K1, EGFR, GSK3B, and CYCS. Transcriptomic validation in GEO datasets prioritized multiple hub genes. In vivo experiments showed activation of apoptosis-related signaling in the brain, while in vitro assays demonstrated ROS accumulation and neuroinflammatory activation (elevated TNF-α, IL-1β, IL-6, IFN-γ). CYCS and MAP2K1 emerged as key convergent nodes. Our findings reveal distinct yet synergistic neurotoxic mechanisms of 6PPD and 6PPD-Q in AD and PD, highlighting tire-derived pollutants as potential environmental risk factors for neurodegenerative diseases.

## 1. Introduction

Globalization and the steady growth of urban traffic have expanded the automotive industry. Tires are indispensable components, and their production and consumption remain high [1]. To improve rubber durability and delay heat- and ozone-driven aging, tire formulations widely use p-phenylenediamine antioxidants. Among them, N-(1,3-dimethylbutyl)-N′-phenyl-p-phenylenediamine (6PPD) is one of the most commonly used and can markedly extend tire service life [1,2,3]. Environmental release of 6PPD is mainly associated with tire–road abrasion, which generates tire and road wear particles (TRWPs) [4,5]. Under atmospheric ozone, 6PPD can be transformed into a more reactive and toxic quinone derivative, 6PPD-quinone (6PPD-Q) [1,3].

The identification of 6PPD-quinone as the primary toxicant responsible for the urban runoff mortality syndrome in coho salmon has underscored the significant ecological threat posed by tire-wear transformation products [2]. With advances in monitoring and analytical methods, 6PPD and 6PPD-Q have been consistently detected in multiple matrices, including road dust, tunnel and ambient particulate matter, stormwater runoff, and surface waters, and their levels show clear spatial associations with major environmental drivers [1,5,6]. For human exposure, ingestion of road or soil dust and direct dermal contact are considered important pathways for 6PPD and its transformation products to enter the body [4,5]. Notably, biomonitoring studies have detected 6PPD-Q or related PPD-Qs in blood, urine, and even cerebrospinal fluid (CSF) [7,8]. In addition, experimental studies in mammals suggest that 6PPD and 6PPD-Q can cause multi-organ toxicity, including pneumonitis, reproductive impairment, and liver injury [9,10,11]. Together, these findings indicate that tire-derived contaminants have become an emerging issue for environmental and public health.

Alzheimer’s disease (AD) and Parkinson’s disease (PD) are the two most burdensome neurodegenerative disorders worldwide. Both progress slowly, involve complex etiologic networks, and still lack curative therapies [12,13,14]. AD is characterized by amyloid-β (Aβ) deposition and tau pathology with synaptic dysfunction [12,14], whereas PD is mainly defined by degeneration of nigrostriatal dopaminergic neurons and α-synuclein pathology [13]. Growing evidence suggests that mitochondrial dysfunction, oxidative stress, neuroinflammation, blood–brain barrier (BBB) disruption, and immunometabolic imbalance are shared processes across AD and PD and may serve as convergence points between environmental exposure and genetic susceptibility [14]. Consistent with this framework, multiple models indicate the neurotoxic and neuroinflammatory potential of 6PPD and 6PPD-Q. For example, zebrafish studies showed that 6PPD can cause cerebrovascular defects accompanied by oxidative stress and ferroptosis-related changes [15], and 6PPD and its metabolites can accumulate in the brain, perturb dopaminergic signaling, and induce locomotor abnormalities [16]. In mammals, long-term exposure to 6PPD-Q can impair cognition or behavior and trigger neuroinflammation. A gut microbiota-dependent mechanism has also been reported, suggesting possible involvement of the gut–brain axis [17]. Importantly, higher levels of 6PPD-Q have been detected in CSF from patients with PD, and 6PPD-Q exacerbated Lewy neurite formation in an α-synuclein preformed fibril (PFF) model via mitochondrial dysfunction, providing a direct human–mechanistic link between 6PPD-Q and PD-related pathology [18].

Despite these advances, the relationship between 6PPD/6PPD-Q exposure and the onset of major neurodegenerative disorders remains a largely unexplored frontier in environmental toxicology. First, most studies focus on single endpoints or pathways and do not capture the multi-pathway, multi-cell-type interaction networks that typify neurodegeneration. Second, whether 6PPD and 6PPD-Q differ in molecular initiating events, key toxic targets, and dominant signaling pathways is unclear. Third, shared targets and disease-specific modules in exposure-response networks between AD and PD have not been directly compared [19,20,21]. Network toxicology integrates systems biology with multi-database evidence to build compound–target–pathway–phenotype and disease interaction networks, enabling identification of hub targets and functional modules that better reflect multi-target, low-dose, and long-term exposure scenarios [19,20]. Combined with protein–protein interaction (PPI) analysis and topology-based ranking, core nodes can be prioritized [22], and molecular docking simulations can be used to validate ligand–receptor binding at the structural level [23,24,25].

Accordingly, this study integrates network toxicology and experimental validation to systematically characterize the neurotoxic interaction networks of 6PPD and 6PPD-Q, with a focus on two major neurodegenerative outcomes, AD and PD. We compare target profiles, key signaling pathways, and shared network nodes between the two compounds to provide a mechanistically interpretable framework for assessing their central nervous system risks and to support future biomarker screening and chemical risk evaluation.

## 2. Results

### 2.1. Toxicity Analysis of 6PPD and 6PPD-Q on Organ Toxicity

To initially assess systemic risks, toxicity predictions were conducted via the ProTox 3.0 platform. In silico evaluations strongly suggested potential neurotoxicity for both 6PPD and 6PPD-Q, highlighting the nervous system as a probable common target organ. Notably, 6PPD also indicated potential respiratory toxicity, whereas 6PPD-Q exhibited a more specific organ-toxicity profile localized within the central nervous system (Table 1). Furthermore, both compounds are predicted to actively cross the blood–brain barrier (BBB), with high probabilities of 0.84 for 6PPD and 0.65 for 6PPD-Q, reinforcing their potential to directly impact neural tissue (Table 2).

### 2.2. The Effects of 6PPD on AD

**Figure 1:**
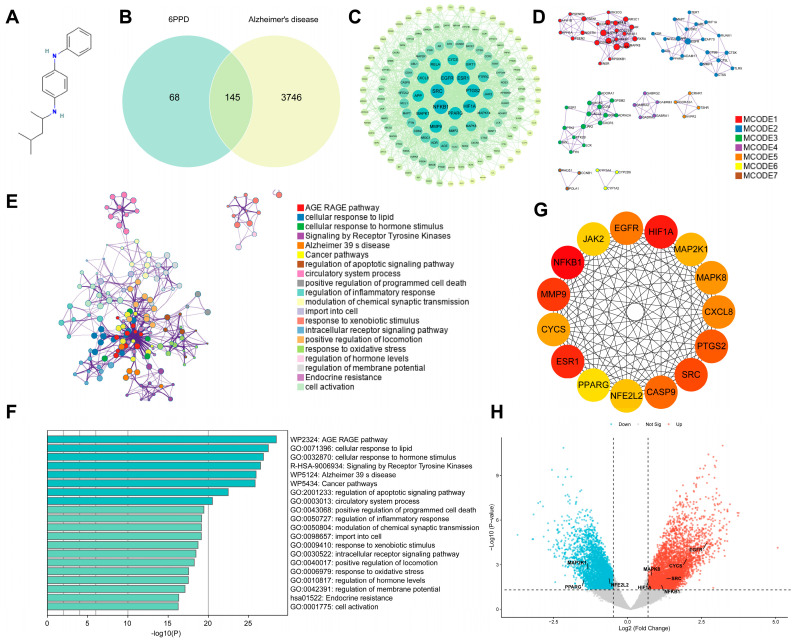
PPI network construction and enrichment analysis of 6PPD-associated targets in AD. (**A**) Two-dimensional chemical structure of 6PPD derived from its canonical SMILES. (**B**) Venn diagram illustrating the intersection of predicted 6PPD targets and AD-associated genes. (**C**) PPI network of the shared targets constructed via the STRING database (confidence score > 0.40). (**D**) Key functional modules extracted from the PPI network using the MCODE algorithm. (**E**) Network visualization of the enriched GO and KEGG pathways for the intersecting targets. (**F**) Bar chart displaying the top significantly enriched functional pathways, ranked by −log10(*p*). (**G**) The top 15 hub genes prioritized using the MCC method. The color gradient indicates the topological essentiality of the nodes. (**H**) Transcriptomic validation of the identified hub genes using the AD clinical dataset GSE5281. The volcano plot highlights the differentially expressed core targets (thresholds: *p* < 0.05 and Fold change > 1.5).

The canonical SMILES string of 6PPD was retrieved from the PubChem database and used to construct its 2D molecular structure (Figure 1A). Based on this chemical information, potential 6PPD-associated targets were collected from four complementary databases, including SwissTargetPrediction, TargetNet, ChEMBL, and STITCH, which together cover predicted targets, curated bioactivity records, and known or inferred chemical–protein interactions. After integration and duplicate removal, 213 potential 6PPD targets were obtained. Concurrently, 3891 AD-associated genes were curated from the GeneCards database to represent the disease-related molecular background of AD. To identify targets that may connect 6PPD exposure with AD-related molecular alterations, the predicted 6PPD targets were overlapped with AD-associated genes. Venn diagram analysis revealed 145 intersection targets shared between 6PPD and AD (Figure 1B). These shared targets were then used as the core input for subsequent network construction. To examine whether these targets formed a functionally connected protein network, a protein–protein interaction (PPI) network was constructed using the STRING database with a confidence threshold of 0.40 (Figure 1C). In this network, nodes represent proteins encoded by the intersecting targets, whereas edges represent known or predicted functional associations. To further explore the internal structure of this PPI network, MCODE-based module enrichment analysis was performed to identify densely connected sub-networks. The analysis showed that 6PPD-associated targets in the AD context were mainly clustered in highly interconnected pathways, particularly regulated proteolysis of p75NTR (R-HSA-193692) and Alzheimer’s disease-specific pathways (WP5124). This module-level result suggests that 6PPD-related targets may converge on neurotrophin-related signaling and AD-associated molecular networks (Table 3). In parallel, whole-gene-set enrichment analysis was performed using all 145 intersecting targets to characterize the broader biological processes and pathways involved in the 6PPD–AD network. This analysis highlighted the AGE-RAGE pathway (WP2324), cellular response to lipid (GO:0071396), and cellular response to hormone stimulus (GO:0032870), suggesting potential involvement of metabolic responses, lipid-related regulation, hormone-mediated signaling, and glycation-stress-associated pathways. In addition, damage-outcome-related terms were significantly enriched, including regulation of the apoptotic signaling pathway (GO:2001233) and positive regulation of programmed cell death (GO:0043068). These findings indicate that 6PPD-associated targets may be linked to oxidative-stress- and inflammation-related processes that converge on apoptosis and programmed cell death, which may be relevant to AD-related tissue injury (Figure 1E,F). Finally, to prioritize key nodes within this interaction network, the maximal clique centrality (MCC) algorithm was applied to rank the top 15 hub genes (Figure 1G). These hub genes were further evaluated using the clinical AD transcriptomic dataset GSE5281. This validation identified nine core targets with statistically significant differential expression, including CYCS, EGFR, HIF1A, MAP2K1, MAPK8, NFE2L2, NFKB1, PPARG, and SRC, as visualized in the volcano plot (Figure 1H).

### 2.3. The Effects of 6PPD on PD

**Figure 2:**
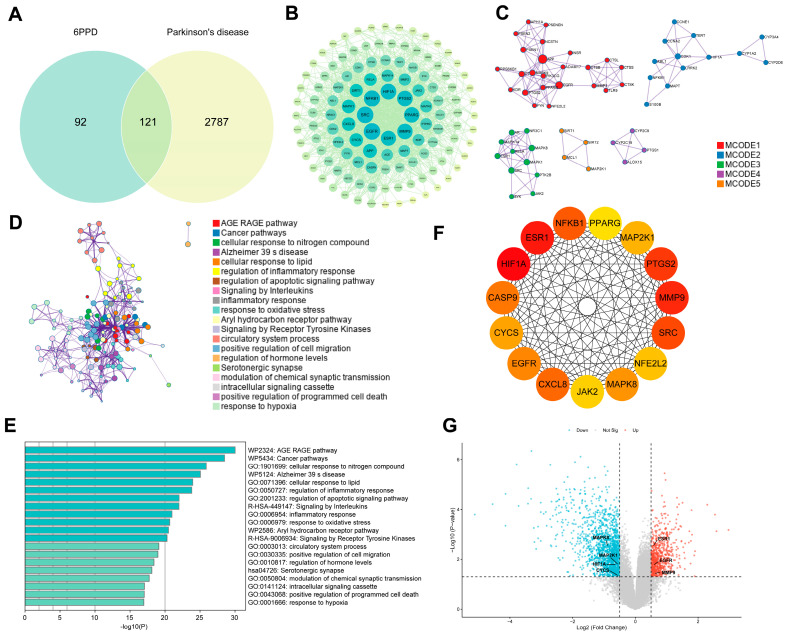
PPI network construction and enrichment analysis of 6PPD-associated targets in PD. (**A**) Venn diagram illustrating the intersection of predicted 6PPD targets and PD-associated genes. (**B**) PPI network of the shared targets constructed via the STRING database (confidence score > 0.40). (**C**) Key functional modules extracted from the PPI network using the MCODE algorithm. (**D**) Network visualization of the enriched GO and KEGG pathways for the intersecting targets. (**E**) Bar chart displaying the top significantly enriched functional pathways, ranked by −log10(*p*). (**F**) The top 15 hub genes prioritized using the MCC method. The color gradient indicates the topological essentiality of the nodes. (**G**) Transcriptomic validation of the identified hub genes using the PD clinical dataset GSE8397. The volcano plot highlights the differentially expressed core targets (thresholds: *p* < 0.05 and Fold change > 1.5).

Expanding our investigation to Parkinson’s disease, 2908 PD-associated genes were retrieved from GeneCards. Intersection analysis identified 121 shared targets linking 6PPD to PD pathology (Figure 2A), which subsequently formed the basis of a PD-specific PPI network (Figure 2B). MCODE topology analysis partitioned this network into tightly connected functional modules. The high-density core module was significantly enriched in the regulated proteolysis of p75NTR (R-HSA-193692) and the PID P75 NTR PATHWAY (M153), suggesting that the perturbation of neuro-receptor processing and its downstream signaling represents an early and prominently affected component in 6PPD-induced neurotoxicity. Another distinct module highlighted the biosynthesis of maresin-like SPMs (R-HSA-9027307), implying a disruptive impact on pro-resolving lipid mediators and the intrinsic mechanisms of inflammation resolution (Table 3). Global functional enrichment in the PD context further emphasized cellular responses to lipids and nitrogen compounds (GO:1901699), suggesting that 6PPD may severely disrupt neuronal homeostasis through concurrent glycation stress and lipid-metabolism alterations. The concurrent enrichment of the regulation of inflammatory response (GO:0050727), response to oxidative stress (GO:0006979), and apoptotic signaling pathways (GO:2001233) establishes a clear mechanistic link, supporting the hypothesis that continuous inflammation and oxidative pressure drive the cell-death processes central to PD pathology (Figure 2D,E). Further validation of the top 15 MCC-ranked hub genes (Figure 2F) against the PD transcriptomic dataset (GSE8397) identified a signature of seven core targets exhibiting significant dysregulation: CYCS, EGFR, ESR1, HIF1A, MAP2K1, MAPK8, and MMP9 (Figure 2G).

### 2.4. Molecular Docking of Core Targets Induced by 6PPD in AD and PD

**Figure 3:**
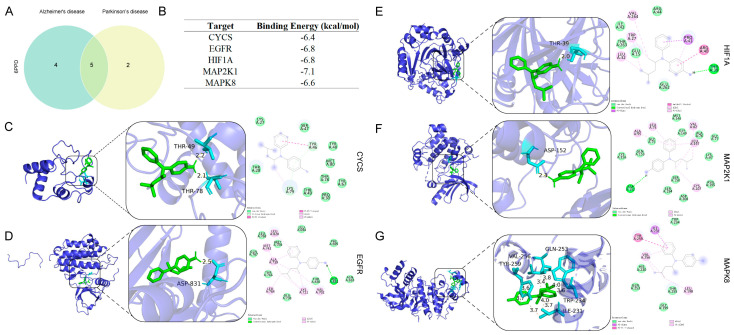
Molecular docking analysis of 6PPD with the shared core targets in AD and PD. (**A**) Venn diagram illustrating the intersection of 6PPD-associated targets with AD and PD, identifying five shared core targets. (**B**) Summary of the binding energies (kcal/mol) between 6PPD and the five shared targets, calculated via AutoDock Vina 1.1.2. (**C**–**G**) Molecular docking simulations demonstrating the optimal binding modes of 6PPD within the active pockets of (**C**) CYCS, (**D**) EGFR, (**E**) HIF1A, (**F**) MAP2K1, and (**G**) MAPK8. Each panel displays the 3D protein–ligand complex (**left**), a magnified 3D view highlighting specific interacting amino acid residues and bond distances (**middle**), and a 2D interaction diagram detailing the types of intermolecular forces, such as hydrogen bonds and hydrophobic contacts (**right**).

To identify shared neurotoxic drivers of 6PPD, we intersected the core targets from both disease models. This analysis pinpointed five overlapping targets including CYCS, EGFR, HIF1A, MAP2K1, and MAPK8, which represent a common molecular bridge between AD and PD (Figure 3A). To computationally evaluate the binding potential and spatial compatibility, molecular docking simulations were meticulously performed for these five shared proteins. All targets exhibited highly favorable binding affinities, with binding energies ranging from −6.4 to −7.1 kcal/mol (Figure 3B). Notably, MAP2K1 exhibited the most robust and stable binding conformation (−7.1 kcal/mol), followed closely by EGFR and HIF1A (both at −6.8 kcal/mol), while MAPK8 (−6.6 kcal/mol) and CYCS (−6.4 kcal/mol) also demonstrated appreciable affinities (Figure 3C–G). The docking simulations predicted favorable binding conformations, characterized by potential hydrogen-bonding networks and hydrophobic interactions. These computational predictions provide a structural hypothesis for the multi-target engagement capability of 6PPD, which warrants further experimental validation.

### 2.5. The Effects of 6PPD-Q on AD

**Figure 4:**
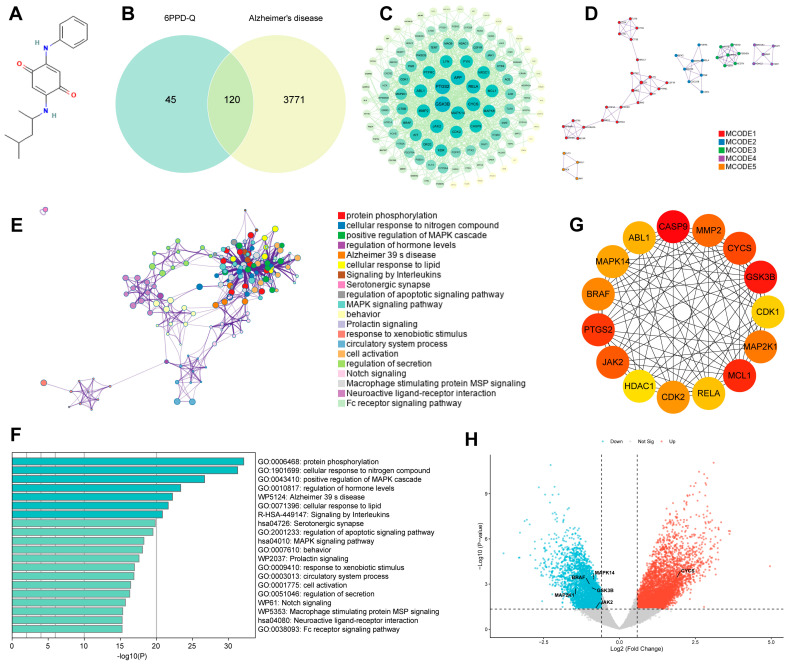
PPI network construction and enrichment analysis of 6PPD-Q-associated targets in AD. (**A**) Two-dimensional chemical structure of 6PPD-Q derived from its canonical SMILES. (**B**) Venn diagram illustrating the intersection of predicted 6PPD-Q targets and AD-associated genes. (**C**) PPI network of the shared targets constructed via the STRING database (confidence score > 0.40). (**D**) Key functional modules extracted from the PPI network using the MCODE algorithm. (**E**) Network visualization of the enriched GO and KEGG pathways for the intersecting targets. (**F**) Bar chart displaying the top significantly enriched functional pathways, ranked by −log10(*p*). (**G**) The top 15 hub genes prioritized using the MCC method. The color gradient indicates the topological essentiality of the nodes. (**H**) Transcriptomic validation of the identified hub genes using the AD clinical dataset GSE5281. The volcano plot highlights the differentially expressed core targets (thresholds: *p* < 0.05 and Fold change > 1.5).

As the highly reactive transformation product of 6PPD, 6PPD-Q was subjected to parallel investigations. Using its canonical SMILES (Figure 4A), 165 predictive targets were compiled. Intersection with the AD-related gene set yielded 120 shared targets (Figure 4B), establishing a dense PPI network (Figure 4C). MCODE-based module enrichment showed that 6PPD-Q-associated targets were clustered in a high-density sub-network enriched for trafficking and processing of endosomal TLRs (R-HSA-1679131) and positive regulation of the MAPK cascade (GO:0043410). These results suggest that innate immune sensing and stress-responsive signaling may be involved in the predicted 6PPD-Q–AD interaction network. Additional modules were enriched in noncanonical activation of NOTCH3 (R-HSA-9017802) and amyloid-beta formation (GO:0034205), indicating a possible association with AD-related protein-processing pathways. (Figure 4D; Table 4). Across the global enriched network, protein phosphorylation (GO:0006468) and the positive regulation of the MAPK cascade (GO:0043410) remained prominent, indicating a massive amplification of kinase-driven stress signaling. Pathways related to cellular responses to nitrogen compounds and xenobiotic stimuli were also enriched, confirming enhanced neuroinflammatory programs (Figure 4E,F). Among the 15 key hub nodes identified by MCC (Figure 4G), six targets including BRAF, CYCS, GSK3B, JAK2, MAP2K1, and MAPK14 were validated in the AD clinical dataset (GSE5281), displaying significant transcriptional dysregulation (Figure 4H).

### 2.6. The Effects of 6PPD-Q on PD

**Figure 5:**
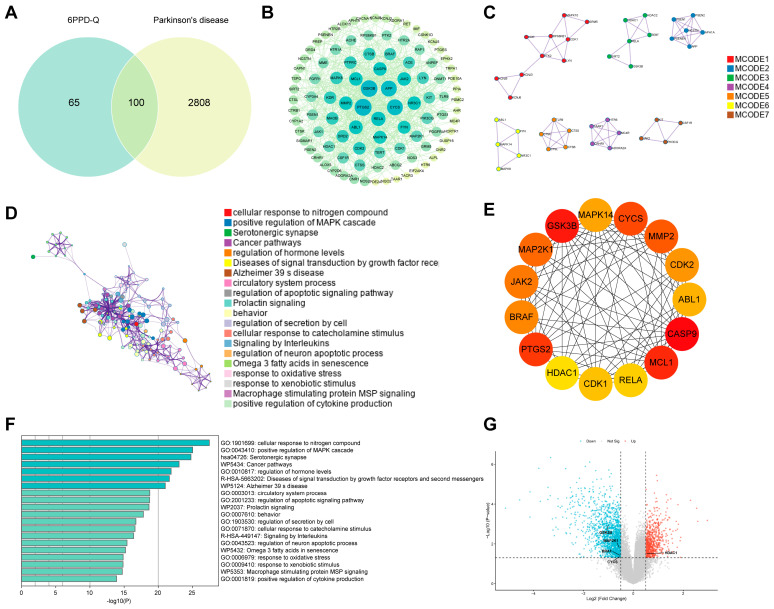
PPI network construction and enrichment analysis of 6PPD-Q-associated targets in PD. (**A**) Venn diagram illustrating the intersection of predicted 6PPD-Q targets and PD-associated genes. (**B**) PPI network of the shared targets constructed via the STRING database (confidence score > 0.40). (**C**) Key functional modules extracted from the PPI network using the MCODE algorithm. (**D**) Network visualization of the enriched GO and KEGG pathways for the intersecting targets. (**E**) Bar chart displaying the top significantly enriched functional pathways, ranked by −log10(*p*). (**F**) The top 15 hub genes prioritized using the MCC method. The color gradient indicates the topological essentiality of the nodes. (**G**) Transcriptomic validation of the identified hub genes using the PD clinical dataset GSE8397. The volcano plot highlights the differentially expressed core targets (thresholds: *p* < 0.05 and Fold change > 1.5).

Intersection analysis identified 100 specific targets shared between 6PPD-Q and PD (Figure 5A), mapping them into a robust PPI network (Figure 5B). MCODE analysis identified a high-density functional module centered on endosomal TLR processing and MAPK cascade regulation, further substantiating the early involvement of innate immune sensing in the biological response. Crucially, additional modules highlighted Oncostatin M signaling (WP2374) and amyloid-beta formation, revealing a dual mechanism of inflammatory regulation and abnormal protein processing in the PD context (Figure 5C; Table 4). Gene-set enrichment further emphasized disruptions in signal transduction pathways driven by growth factor receptors and second messengers (R-HSA-5663202). The prominent enrichment of oxidative stress responses, xenobiotic stimulus responses, and interleukin signaling synergistically indicates that 6PPD-Q heavily exacerbates PD-related neuronal injury through inflammatory amplification and targeted apoptosis (Figure 5D,E). Following MCC-based prioritization (Figure 5F), transcriptomic validation using the GSE8397 dataset pinpointed five core targets, specifically BRAF, CYCS, GSK3B, HDAC1, and MAP2K1, that displayed significant transcriptional dysregulation (Figure 5G).

### 2.7. Molecular Docking of Core Targets Induced by 6PPD-Q in AD and PD

**Figure 6:**
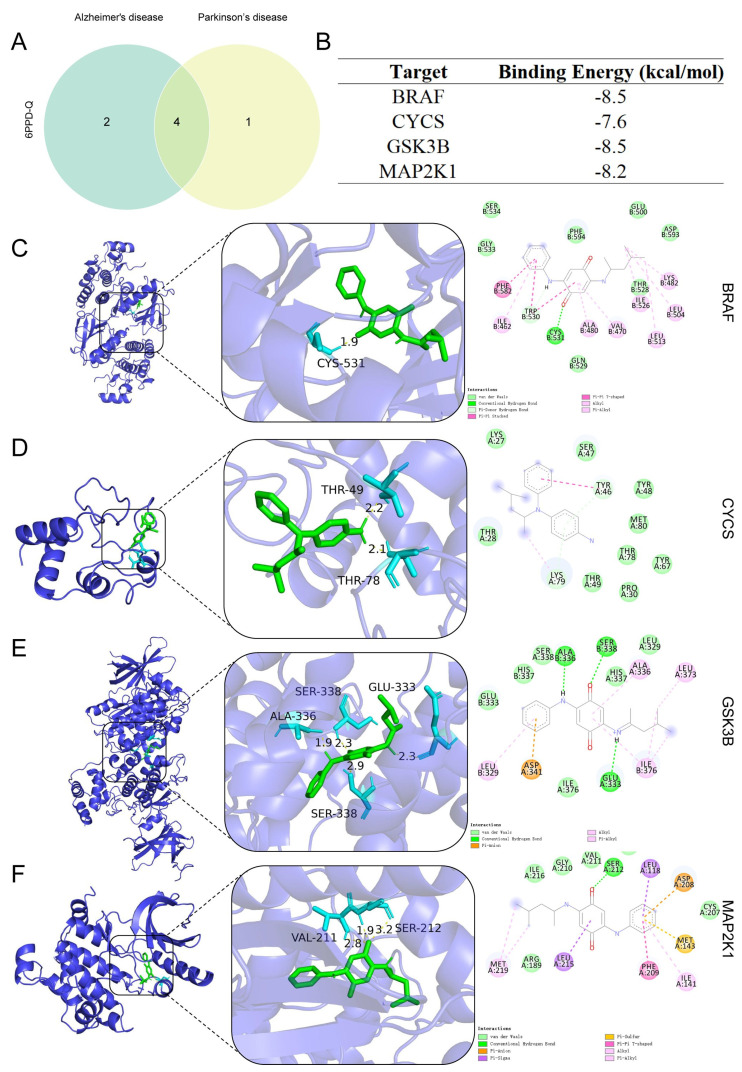
Molecular docking analysis of 6PPD-Q with the shared core targets in AD and PD. (**A**) Venn diagram illustrating the intersection of 6PPD-Q-associated targets with AD and PD, identifying four shared core targets. (**B**) Summary of the binding energies (kcal/mol) between 6PPD-Q and the four shared targets, calculated via AutoDock Vina. (**C**–**F**) Molecular docking simulations demonstrating the optimal binding modes of 6PPD within the active pockets of (**C**) BRAF, (**D**) CYCS, (**E**) GSK3B, and (**F**) MAP2K1. Each panel displays the 3D protein–ligand complex (**left**), a magnified 3D view highlighting specific interacting amino acid residues and bond distances (**middle**), and a 2D interaction diagram detailing the types of intermolecular forces, such as hydrogen bonds and hydrophobic contacts (**right**).

Intersectional analysis revealed a signature of four absolute core targets (BRAF, GSK3B, MAP2K1, and CYCS) representing convergent molecular vulnerabilities to 6PPD-Q across both neurodegenerative diseases (Figure 6A). Docking simulations revealed exceptionally robust binding affinities, with all four targets surpassing the threshold of −7.6 kcal/mol (Figure 6B). BRAF and GSK3B displayed the strongest binding potential (both at −8.5 kcal/mol), followed by MAP2K1 (−8.2 kcal/mol) and CYCS (−7.6 kcal/mol). Structural inspection revealed stable hydrogen bonds and extensive hydrophobic interactions locking the ligand within the binding pockets, which may support the potential binding of 6PPD-Q to these neurodegenerative hubs (Figure 6C–F).

### 2.8. Integrative Analysis of the Core Targets for 6PPD and 6PPD-Q

**Figure 7:**
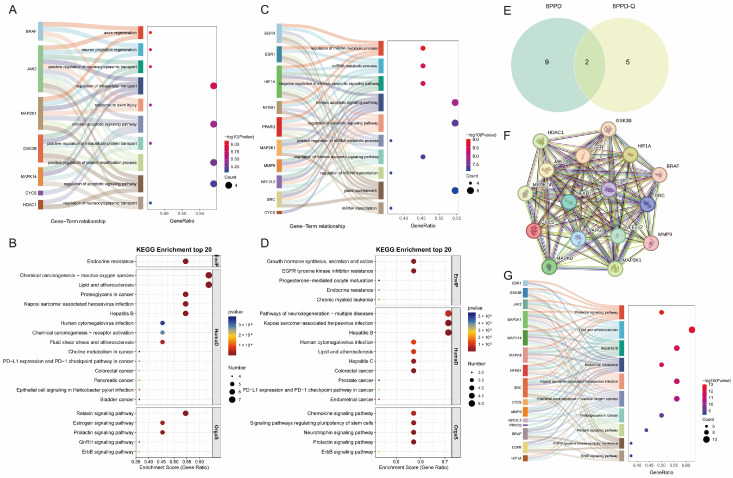
Comprehensive analysis of union core targets of 6PPD and 6PPD-Q. (**A**) GO analysis and (**B**) KEGG pathway enrichment analysis of the 6PPD union core targets. (**C**) GO analysis and (**D**) KEGG pathway enrichment analysis of the 6PPD-Q union core targets. (**E**) Venn diagram illustrating the intersection of neurodegeneration-related core targets between 6PPD and 6PPD-Q, identifying two shared targets. (**F**) PPI network of the combined core targets highlighting the dense connectivity among key hub genes. (**G**) Gene-pathway relationship plot illustrating the core KEGG pathways synergistically enriched by the combined targets. In all enrichment plots, node size represents the gene count, and the color gradient indicates statistical significance −log10(*p*).

To systematically delineate the mechanistic divergences and commonalities between the parent compound and its quinone derivative, comparative pathway enrichment was performed. GO enrichment analysis indicated that the predicted target networks of both compounds were associated with the intrinsic apoptotic signaling pathway, while their upstream enriched biological processes differed. The predicted 6PPD target network showed enrichment in “axon regeneration” and “response to axon injury,” suggesting a possible association with axonal repair-related processes. In contrast, the 6PPD-Q target network was enriched in “miRNA metabolic process,” with HIF1A and NFKB1 connected to intrinsic apoptosis-related terms, suggesting potential involvement of post-transcriptional regulation and cell-death-related pathways. (Figure 7A,B). KEGG pathway mapping revealed a common landscape of metabolic-immune disruptions including endocrine resistance, lipid and atherosclerosis pathways, and the PD-L1 checkpoint pathway. KEGG enrichment analysis further suggested compound-specific pathway patterns: 6PPD-associated targets were enriched in oxidative stress-related pathways, including chemical carcinogenesis and reactive oxygen species pathways, whereas 6PPD-Q-associated targets were enriched in neurodegeneration-related, chemokine-related, and neurotrophin-related signaling pathways. (Figure 7C,D). Finally, intersection analysis of the overall neurotoxicity networks identified CYCS and MAP2K1 as the absolute, shared core nodes between 6PPD and 6PPD-Q (Figure 7E). The dense PPI sub-network formed by these combined targets, featuring highly connected hubs like EGFR, JAK2, MAPK14, NFKB1, PPARG, and HIF1A (Figure 7F), ultimately maps to overarching pathways such as RTK or MAPK signaling and lipid-driven inflammation (Figure 7G). These integrative results suggest that oxidative stress- and inflammation-related pathways may represent potential convergent processes linking 6PPD and 6PPD-Q exposure to neurodegeneration-related molecular networks.

### 2.9. In Vivo Experimental Validation of 6PPD and 6PPD-Q Neurotoxicity

**Figure 8:**
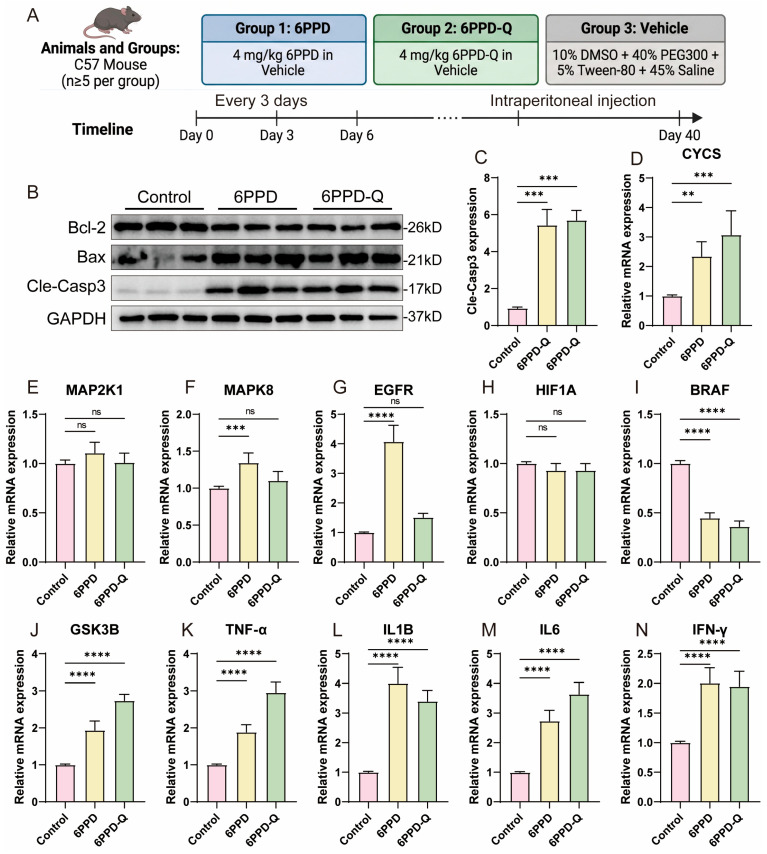
In vivo validation of 6PPD and 6PPD-Q-induced neurotoxicity in C57BL/6 mice. (**A**) Schematic representation of the in vivo experimental design. C57BL/6 mice (*n* = 5 per group) were intraperitoneally injected with vehicle, 6PPD (4 mg/kg), or 6PPD-Q (4 mg/kg) every 3 days for 40 days. (**B**) Representative Western blot images of apoptosis-related proteins (Bcl-2, Bax, and Cle-Casp3) in the brain tissue. (**C**) Statistical quantification of relative Cle-Casp3 protein expression. (**D**–**J**) Relative mRNA expression levels of predicted core targets in the brain tissue measured by RT-qPCR: (**D**) CYCS, (**E**) MAP2K1, (**F**) MAPK8, (**G**) EGFR, (**H**) HIF1A, (**I**) BRAF, and (**J**) GSK3B. (**K**–**N**) Relative mRNA expression levels of pro-inflammatory cytokines measured by RT-qPCR: (**K**) TNF-α, (**L**) IL-1β, (**M**) IL-6, and (**N**) IFN-γ. Data are presented as mean ± SD. ** *p* < 0.01, *** *p* < 0.001, **** *p* < 0.0001; ns, not significant.

To validate the in silico neurotoxicity predictions, an in vivo exposure model was established wherein C57BL/6 mice were intraperitoneally injected with vehicle, 6PPD, or 6PPD-Q over a 40-day period (Figure 8A). Western blot analysis of whole-brain samples (Figure 8B) and subsequent protein quantification (Figure 8C) showed that both toxicants activated apoptosis-related signaling, as evidenced by the marked upregulation of cleaved-caspase 3. RT-qPCR was then utilized to assess the mRNA expression of the computationally prioritized core targets. CYCS expression was significantly elevated by both compounds (Figure 8D). MAP2K1 exhibited no significant transcriptional changes at this specific time point (Figure 8E). MAPK8 and EGFR were significantly upregulated, predominantly following 6PPD exposure (Figure 8F,G), while HIF1A expression remained unaltered across all groups (Figure 8H). Conversely, BRAF mRNA was significantly downregulated by both toxicants (Figure 8I), whereas GSK3B was significantly upregulated (Figure 8J). Finally, corroborating the predicted acceleration of severe neuroinflammatory cascades, both 6PPD and 6PPD-Q triggered a profound inflammatory response in the brain tissue. This was characterized by highly significant elevations in the mRNA levels of pro-inflammatory cytokines, specifically TNF-α (Figure 8K), IL-1β (Figure 8L), IL-6 (Figure 8M), and IFN-γ (Figure 8N).

### 2.10. In Vitro Validation of 6PPD and 6PPD-Q Induced Oxidative Stress and Neuroinflammation

**Figure 9:**
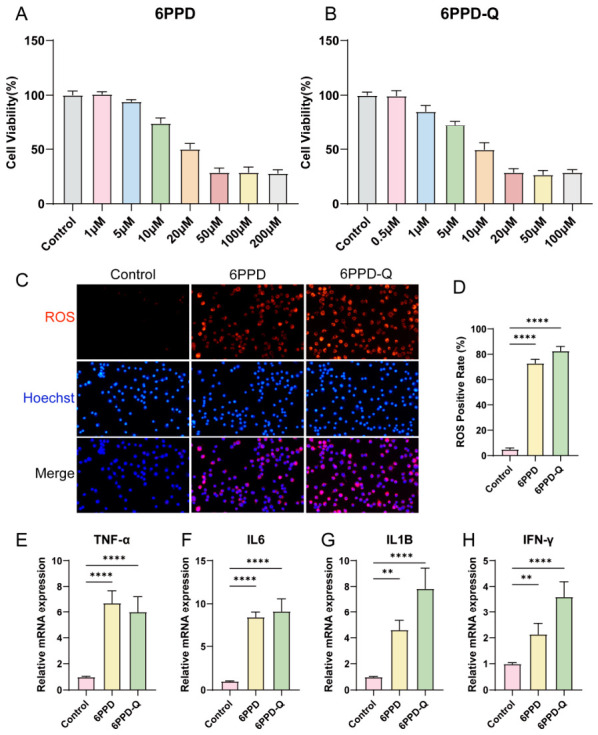
6PPD and 6PPD-Q drive cytotoxicity, ROS accumulation, and pro-inflammatory cytokine expression in BV2 microglia. (**A**) Cell viability of BV2 cells treated with varying concentrations of 6PPD (1–200 μM). (**B**) Cell viability of BV2 cells treated with varying concentrations of 6PPD-Q (0.5–100 μM). (**C**) Representative fluorescence images of intracellular ROS generation. Red fluorescence indicates ROS accumulation, and blue fluorescence (Hoechst) indicates cell nuclei. (**D**) Quantitative analysis of the ROS positive rate (%). (**E**–**H**) Relative mRNA expression levels of pro-inflammatory cytokines in BV2 cells measured by RT-qPCR: (**E**) TNF-α, (**F**) IL-6, (**G**) IL-1β, and (**H**) IFN-γ. Data are presented as mean ± SD from independent biological triplicates (*n* = 4 per group). For (**C**–**H**), BV2 cells were treated with 5 µM 6PPD or 1 µM 6PPD-Q for ROS detection and RT-qPCR analysis. ** *p* < 0.01, **** *p* < 0.0001.

To validate the in silico predictions of oxidative stress and neuroinflammation, two well-established microglial functions in AD and PD pathogenesis, we exposed murine BV2 microglia to 6PPD and 6PPD-Q. Cell viability assays confirmed that both 6PPD (Figure 9A) and 6PPD-Q (Figure 9B) induced dose-dependent cytotoxicity. Validating the predicted induction of oxidative stress, fluorescence imaging and subsequent quantification demonstrated a massive and significant intracellular accumulation of reactive oxygen species (ROS) in both treatment groups (Figure 9C,D). Furthermore, RT-qPCR analysis corroborated the computational prediction of severe pro-inflammatory cascades. Both compounds triggered profound neuroinflammatory activation, characterized by highly significant mRNA upregulations of TNF-α (Figure 9E), IL-6 (Figure 9F), IL-1β (Figure 9G), and IFN-γ (Figure 9H). Consistent with its predicted profile as an aggressive pharmacological hijacker, 6PPD-Q elicited a notably stronger upregulation of IL-1β and IFN-γ compared to 6PPD.

## 3. Discussion

Environmental chemical exposures are widely acknowledged as risk factors for oxidative stress and neuroinflammation. However, the upstream molecular targets initiating these neurodegenerative cascades are often difficult to identify. The ubiquitous tire rubber antioxidant 6PPD and its atmospheric transformation product 6PPD-Q present emerging ecological and public health hazards. Yet, their specific multi-target neurotoxic mechanisms regarding Alzheimer’s disease and Parkinson’s disease remain largely unexplored [26,27]. To address this gap, we integrated network toxicology, structural pharmacology, and biological experimental validations. Our findings indicate that 6PPD and 6PPD-Q exert distinct yet interconnected neurotoxic effects. They act synergistically to drive core hallmarks of neurodegenerative diseases. By translating theoretical binding interactions into validated pathological mechanisms, this study offers a functional framework for evaluating the central nervous system risks associated with tire wear pollutants.

A fundamental requirement for any neurotoxicant is its capacity to enter the brain parenchyma. Our initial computational profiling established this physiological baseline. Both compounds showed a high predicted probability of crossing the blood–brain barrier, scoring 0.84 for 6PPD and 0.65 for 6PPD-Q. The lipophilic nature of these molecules facilitates structural compatibility with the lipid bilayers of endothelial cells. This penetrability supports their ability to infiltrate the brain, accumulate in lipid-rich neural tissues, and directly trigger localized neurotoxicity [28]. Our animal experiments provided clear biological confirmation. Following intraperitoneal administration in C57BL/6 mice, we observed severe apoptotic and inflammatory alterations directly within the brain tissues. This demonstrates that these toxicants successfully breach systemic defenses to cause significant structural and functional damage in the central nervous system.

Through comparative network profiling and experimental validation, we found that 6PPD and 6PPD-Q operate via temporally and spatially distinct mechanisms. The parent compound 6PPD primarily acts as a foundational disruptor of neuronal structural integrity and oxidative homeostasis. Functional enrichment analyses indicated that 6PPD mediated toxicity is heavily biased toward disrupting axon regeneration and the AGE-RAGE signaling pathway. In the adult brain, maintaining extensive axonal networks is essential for synaptic plasticity [29]. Furthermore, 6PPD targets clustered significantly in the regulated proteolysis of p75NTR. This neurotrophin receptor orchestrates the balance between neuronal survival and proneurotrophin induced apoptosis [30]. In vitro validation using BV2 microglia supported this foundational disruption hypothesis. Exposure to 6PPD induced a massive and dose-dependent accumulation of intracellular reactive oxygen species. Concurrently, in vivo transcriptomic data showed significant upregulations of EGFR and MAPK8 in the brains of 6PPD-exposed mice. EGFR signaling is implicated in reactive astrogliosis, while MAPK8 is a classic stress-responsive kinase that initiates cell death under oxidative pressure [31,32]. We therefore propose that chronic low-dose 6PPD exposure acts as a pervasive initial insult. It systematically erodes neuronal resilience and establishes a toxic microenvironment characterized by unresolving glycation stress and lipid peroxidation.

The toxicological profile of the ozonation product 6PPD-Q presents a highly aggressive intervention into the definitive proteinopathic hallmarks of neurodegenerative diseases. Network analysis revealed that 6PPD-Q targets are specifically enriched in canonical pathways of neurodegeneration and are intimately linked to amyloid beta formation and endosomal Toll-like receptor processing. This aggressiveness was clearly illustrated by our thermodynamic molecular docking simulations. 6PPD-Q demonstrated exceptionally stable binding to neurodegenerative kinase hubs, notably GSK3B and BRAF, with both achieving binding energies of −8.5 kcal/mol. However, simulations predicted favorable binding conformations; future kinase activity assays are required to determine whether these interactions result in inhibition or activation. GSK3B is a recognized pathological linchpin in both Alzheimer’s and Parkinson’s diseases. It regulates the hyperphosphorylation of tau proteins and modulates amyloid precursor protein cleavage [33,34]. Our animal data validated this prediction, showing a significant upregulation of GSK3B mRNA in the brains of exposed mice. By directly hijacking this kinase network, 6PPD-Q bypasses peripheral stress responses to actively accelerate the formation of neurotoxic protein aggregates. Although not directly measured in this study, our pathway enrichment results suggest that 6PPD-Q may be potentially involved in pathways related to amyloid-β formation (GO:0034205). Whether this translates into actual protein aggregation or tau pathology requires direct experimental validation in future studies.

This pharmacological hijacking extends deeply into the immune architecture of the brain. Endosomal Toll-like receptors play an indispensable role in innate immune sensing within microglia [35]. Overactivation of these receptors by xenobiotic stimuli initiates an overwhelming cascade of proinflammatory cytokines. This microglial cytokine storm chronically poisons the surrounding neuronal milieu and accelerates neurodegeneration [36,37]. Our experimental data provide strong evidence of this phenomenon. Exposure to 6PPD-Q triggered profound neuroinflammatory activation in BV2 microglia, characterized by the aggressive upregulation of IL-1 beta and IFN gamma. Similarly, brain tissues from exposed mice exhibited massive surges in TNF alpha, IL-6, and IL-1 beta transcripts. The localized overproduction of these cytokines induces direct excitotoxicity. It also creates an autocrine and paracrine loop that further activates surrounding glia, leading to a self-sustaining cycle of neuroinflammation typical of progressing neurodegenerative diseases [38].

Despite initiating toxicity through different upstream receptors and kinases, the global neurotoxicity profiles of both compounds share a convergent execution pathway. Intersection analysis identified CYCS and MAP2K1 as the absolute shared core nodes between 6PPD and 6PPD-Q. MAP2K1 acts as an irreplaceable central transducer within the MAPK signaling cascade. It funnels and amplifies diverse upstream inflammatory stimuli down into the nucleus [39]. Simultaneously, the dysregulation and cytosolic release of cytochrome c serve as definitive biomarkers of unrecoverable mitochondrial dysfunction [40,41]. This marks the irreversible execution phase of intrinsic apoptosis via apoptosome formation. Our experimental models successfully recapitulated this computational prediction. In the mouse model, we observed a significant elevation of CYCS mRNA across both exposure groups. Western blot analyses of the brain tissues demonstrated a marked induction of apoptosis, evidenced by the severe upregulation of Cleaved-Caspase 3 and disruptions in the Bax to Bcl-2 ratio. This confirms that the toxicological trajectories of both 6PPD and 6PPD-Q inevitably converge on catastrophic mitochondrial apoptosis-related pathways.

Based on this topological and experimental convergence, we propose a synergistic neurotoxic model for tire-derived pollutants. Under real-world environmental exposure, the human central nervous system is rarely subjected to these chemicals in isolation. Instead, it encounters them as a dynamic mixture. In our pathological model, the parent compound 6PPD administers a chronic initial hit. It induces lipid-driven oxidative stress, impairs the capacity for axonal regeneration, and establishes a baseline of neuronal vulnerability. Following this structural weakening, the highly reactive quinone derivative 6PPD-Q capitalizes on the compromised cellular state. It delivers a catastrophic second hit by directly binding to and aberrantly activating kinases like GSK3B. Although whole-brain samples and BV2 microglia were used to assess apoptosis-related signaling, oxidative stress, and neuroinflammation, neuronal in vitro models were not included. Future studies using SH-SY5Y cells, HT22 cells, primary neurons, or neuron–microglia co-cultures are needed to clarify direct neuronal responses to 6PPD and 6PPD-Q. This direct pharmacological interference unleashes an uncontrollable proinflammatory cytokine storm from resident microglia, accelerates pathological protein aggregation, and ultimately precipitates the fatal release of cytochrome c, culminating in neuronal death.

While this research provides a biologically validated mechanistic framework, certain limitations must be acknowledged. Our molecular docking offers biophysical evidence of ligand receptor affinities, but these represent static structural snapshots. Future computational efforts should incorporate long timescale all atom molecular dynamics simulations to ascertain the thermodynamic conformational stability of 6PPD-Q within complex multidomain proteins like GSK3B. Furthermore, while our animal experiments used *n* = 5 per group based on a priori power analysis for large-effect endpoints (apoptosis, inflammation), we acknowledge that this sample size may not be sufficient to detect smaller effect sizes or to fully capture inter-individual variability in neurodegenerative phenotypes. Independent replication in larger cohorts is warranted. Beyond sample size considerations, the current study also lacks direct assessment of AD/PD-specific proteinopathies. The precise spatiotemporal mapping of tau hyperphosphorylation and amyloid plaque deposition requires specialized transgenic animal models subjected to long-term chronic exposures. Evaluating these mechanisms in primary human Alzheimer’s or Parkinson’s brain samples post mortem will provide the ultimate validation of this environmental pathological link. Additionally, our in vitro data primarily support generalized neurotoxic and pro-inflammatory effects, whereas conclusions regarding AD/PD-specific proteinopathies rely on in silico predictions, transcriptomic datasets, and in vivo brain tissue analyses. Future studies should incorporate neuronal cell lines (e.g., SH-SY5Y, primary cortical neurons), dopaminergic models, or co-culture systems to directly validate the effects of 6PPD and 6PPD-Q on amyloid-beta pathology and α-synuclein aggregation. Nonetheless, the current study demonstrates that the ubiquitous tire-derived pollutants 6PPD and 6PPD-Q are potent synergistic drivers of neurodegeneration, emphasizing the necessity for safer industrial chemical alternatives.

## 4. Materials and Methods

### 4.1. In Silico Toxicological Profiling

The canonical SMILES strings for 6PPD and 6PPD-Q were retrieved from the PubChem database and submitted to the ProTox 3.0 platform for in silico toxicological profiling. Organ-specific toxicity and other toxicological endpoints were predicted using the built-in ProTox 3.0 computational models, which integrate chemical-structure information, chemical similarity, and machine-learning-based prediction algorithms trained on curated toxicological datasets. For each endpoint, the platform provides a predicted classification, such as active or inactive, together with a model-reported probability/confidence score. The prediction classes and probability values reported in Table 1 and Supplementary Table S2 were directly obtained from the ProTox 3.0 output. These values therefore represent the confidence scores for the corresponding predicted classifications, rather than experimentally measured probabilities or results generated from an additional statistical model constructed in this study. The ProTox 3.0 results were used as preliminary in silico screening evidence and were interpreted together with subsequent target prediction, network toxicology, molecular docking, transcriptomic validation, and experimental assays.

### 4.2. Compilation of Targets of 6PPD and 6PPD-Q

To initiate compound target prediction, 6PPD and 6PPD-Q were queried separately in four computational platforms: SwissTargetPrediction, TargetNet, ChEMBL, and STITCH. The query inputs were based on compound-level chemical information rather than disease-related keywords. As summarized in Supplementary Table S1, the PubChem-derived canonical SMILES strings of 6PPD and 6PPD-Q were used as the primary inputs for SwissTargetPrediction and TargetNet. For ChEMBL, target information was retrieved using canonical SMILES together with compound-name-based queries, including “6PPD,” “N-(1,3-dimethylbutyl)-N′-phenyl-p-phenylenediamine,” “6PPD-Q,” and “6PPD-quinone.” For STITCH, chemical–protein interaction targets were retrieved using compound names, available chemical identifiers, and PubChem-derived canonical SMILES when applicable. The organism was restricted to *Homo sapiens* whenever species filtering was available. The predicted or retrieved targets from the four platforms were pooled, duplicate entries were removed, and a unique set of candidate proteins was generated. To resolve nomenclature discrepancies, the UniProtKB ID Mapping service was used to convert all preliminary target identifiers into standardized official gene symbols. This curated target set was used as the candidate target library for subsequent network analyses.

### 4.3. Identification of Target Networks Associated with Neurodegenerative Diseases

To explore the specific neurotoxic mechanisms of 6PPD and 6PPD-Q, this study focused on two major neurodegenerative diseases: AD and PD. Disease-associated targets for AD and PD were independently retrieved from the GeneCards Database. Only genes with a relevance score ≥ 5.0 were included. The UniProt ID mapping service was used to standardize gene symbols. Duplicate targets from different databases were removed before Venn diagram analysis. The overlapping targets between each compound and each disease were defined as potential neurotoxicity targets.

### 4.4. Construction of Protein Interaction Network and Screening of Hub Targets

To elucidate the molecular interactions and biological pathways mediating the neurotoxicity of 6PPD and 6PPD-Q, we independently constructed PPI networks for the AD and PD intersection target sets using the STRING database (http://string-db.org). For both networks, the organism was restricted to *Homo sapiens*, and a minimum required interaction score of 0.40 was applied. The constructed networks were visualized and analyzed in Cytoscape (version 3.10.3), where key topological features, including degree centrality, closeness centrality, and betweenness centrality, were assessed. Core potential targets for both AD and PD were identified using the cytoHubba plug-in, applying the MCC algorithm. MCC was selected due to its proven ability to identify biologically meaningful hub genes by scoring nodes based on their involvement in multiple maximum cliques, which outperforms simpler metrics in complex biological networks.

### 4.5. Analysis of Target Protein Function and Pathway Enrichment

Functional enrichment (GO and KEGG) analysis was performed for the distinct AD and PD target sets using Metascape (version 3.5). All enrichment results were corrected for multiple testing using the Benjamini–Hochberg false discovery rate (FDR) method. A term was considered statistically significant if FDR-adjusted *p* < 0.05 and the minimum number of overlapping genes was ≥3. Furthermore, the molecular complex detection (MCODE) algorithm was applied to detect densely connected network modules, which often represent critical protein complexes linked to neurodegenerative processes and neuroinflammation. The findings were visualized using network diagrams and bar plots, highlighting the specific neurotoxic pathways and molecular alterations associated with 6PPD and 6PPD-Q exposure in the context of AD and PD.

### 4.6. Screening of Key Neurotoxicity Targets via Transcriptomic Validation

To enhance the predictive accuracy of core neurotoxicity targets, the GEO database was utilized to download clinical transcriptomic datasets related to AD and PD. Specifically, the dataset GSE5281 was selected for AD, while GSE8397 was retrieved for PD. Detailed characteristics of these two datasets are provided in Supplementary Table S2. The expression profiles of potential targets for 6PPD and 6PPD-Q were independently analyzed within these specific disease cohorts. For microarray data (GSE5281), raw CEL files were normalized using the Robust Multiaverage (RMA) algorithm implemented in the ‘affy’ R package. For GSE8397 (RNA-seq), normalized count data were obtained directly from GEO. Differential expression analysis was performed using the ‘limma’ package (for microarray) and ‘DESeq2’ (for RNA-seq). Differentially expressed genes (DEGs) were defined as |log_2_ fold change| ≥ 0.585 (equivalent to fold change ≥ 1.5 or ≤0.67) and *p*-value < 0.05. By intersecting these DEGs with our previously established AD and PD network targets, we identified the potential functional targets for 6PPD and 6PPD-Q, designating them as the core neurotoxicity targets. Finally, the ggplot2 package in R was employed to construct volcano plots, distinctively annotating these core targets to facilitate the visual interpretation of their expression patterns.

### 4.7. Molecular Docking

Molecular docking was performed using AutoDock Vina (v1.1.2) to evaluate the binding affinities between active compounds and key targets. Protein preparation, including ligand separation, dehydration, and heteroatom removal, was conducted via PyMOL (v4.3.0), while AutoDockTools (v1.5.7) was employed for adding hydrogen atoms, calculating charges, and defining the docking grid box and rotatable bonds. All structures were converted to PDBQT format for semi-flexible docking simulations. Post-docking interactions were visualized in 3D using PyMOL and 2D via Discovery Studio (v19.1.0) . A binding energy < 0 kcal/mol indicates spontaneous binding, while a value < −5.0 kcal/mol signifies a stable and strong protein–ligand interaction.

### 4.8. In Vivo Exposure Model

The ethical approval for all animal experiments was granted by the Institutional Animal Care and Use Committee at Central South University. We utilized healthy, eight-week-old male C57BL/6 mice. The animals were randomly assigned to three experimental cohorts using a random-number-based allocation method (*n* = 5 per cohort): a vehicle-treated control, a 6PPD-treated group (4 mg/kg), and a 6PPD-Q-treated group (4 mg/kg). To reduce potential bias, animal cages and collected tissue samples were labeled with coded identifiers. Investigators responsible for Western blot quantification and RT-qPCR analysis were blinded to treatment allocation during data acquisition and primary quantification. Group information was decoded only after the initial analysis was completed. The dose of 4 mg/kg was selected based on previous mammalian toxicology and toxicokinetic studies of 6PPD and 6PPD-Q [42,43]. In C57BL/6 mice, 4 mg/kg 6PPD or 6PPD-Q has been used to investigate urinary excretion, organ distribution, placental transfer, and developmental exposure-related risks. For a consecutive period of 40 days, the respective treatments were administered via intraperitoneal injection at three-day intervals. Animal body weights were monitored continuously prior to each dose. After 40 days, mice were anesthetized with 1% pentobarbital sodium (70 mg/kg) for deep anesthesia. Once the absence of pedal withdrawal reflex was confirmed, whole brain tissues were harvested for downstream molecular evaluations. Male mice were selected to reduce sex-related biological variability, including potential effects of hormonal cycling, and to maintain consistency across treatment groups in this preliminary mechanistic validation study.

### 4.9. Cell Culture

The BV2 cells were maintained in Dulbecco’s Modified Eagle Medium (DMEM). The culture medium was enriched with 10% fetal bovine serum (FBS) and an antibiotic mixture containing 100 U/mL penicillin and 100 μg/mL streptomycin. All cells were incubated in a standard humidified environment supplied with 5% CO_2_ at 37 °C.

### 4.10. Cell Viability Assessment

To evaluate cellular metabolic activity, BV2 cells were plated in 96-well microplates (3 × 10^3^ cells/well) and incubated overnight to permit attachment. Subsequently, the cells were exposed to a gradient of 6PPD or 6PPD-Q concentrations for a duration of 48 h. Following treatment, cell viability was quantified utilizing the Cell Counting Kit-8 (Beyotime Biotechnology, Shanghai, China). In brief, the CCK-8 reagent was introduced into each well, followed by incubation at 37 °C. The optical density (OD) was ultimately recorded at a wavelength of 450 nm using a microplate spectrophotometer.

### 4.11. Western Blot

Total proteins from brain tissues were extracted using RIPA buffer containing protease and phosphatase inhibitors (Roche, Risch-Rotkreuz, Switzerland) and quantified via a BCA assay (Thermo Fisher Scientific, Waltham, MA, USA). Equal protein amounts (20 μg/lane) were resolved by SDS-PAGE and transferred to PVDF membranes (Millipore, Burlington, MA, USA). After blocking with 5% skim milk in TBST for 1 h at room temperature, membranes were incubated overnight at 4 °C with primary antibodies against Bcl-2, Bax, Cle-Casp3, and GAPDH (Abclonal, Wuhan, China). Following TBST washes, membranes were probed with HRP-conjugated secondary antibodies for 1 h at room temperature. Protein bands were visualized using an ECL system and quantified with ImageJ (v1.53) , with expression levels normalized to GAPDH.

### 4.12. Quantitative Real-Time PCR (RT-qPCR)

Total RNA was extracted using the FastPure^®^ Cell/Tissue Total RNA Isolation Kit (Vazyme, Nanjing, China). cDNA was synthesized using the TransScript All-in-One First-Strand cDNA Synthesis SuperMix (TransGen, Beijing, China). qPCR was performed on a Roche LightCycler 480 using TransScript Probe One-Step qRT-PCR SuperMix. The housekeeping gene was β-actin, which was confirmed to have stable expression across all treatment groups (Ct variation < 0.5 cycles). All primer sequences, amplicon lengths, and annealing temperatures are provided in Supplementary Table S3. Amplification efficiencies for each primer pair were calculated from standard curves (10-fold serial dilutions of cDNA) and ranged from 90% to 110%. Relative gene expression was calculated using the 2^−ΔΔCt^ method.

### 4.13. ROS Detection Assay

Intracellular ROS levels were detected using the CellROX^®^ Red Reagent (Invitrogen, Carlsbad, CA, USA). Post-exposure, BV2 cells underwent two washes with warm PBS, followed by a 30-min incubation with the ROS probe (1 μM) in serum-free DMEM at 37 °C under light-protected conditions. Unbound dye was then eliminated via three consecutive PBS washes. Nuclear counterstaining was achieved by applying Hoechst 33342 (1 μg/mL) for 5 min. The generation of ROS, indicated by red fluorescent signals, was then visualized and recorded utilizing a fluorescence microscope.

### 4.14. Statistical Analysis

All quantitative results are derived from a minimum of three independent biological experiments and are expressed as the mean ± SD. Before statistical analysis, the raw data were reviewed for potential outliers and technical anomalies. No data points were excluded from the final analyses. Statistical evaluations were performed utilizing GraphPad Prism (version 10.1.2). To determine significance between two specific groups, a two-tailed unpaired Student’s *t*-test was applied. For variance across multiple experimental groups, a one-way analysis of variance (ANOVA) was utilized. Statistical significance was established at a threshold of *p* < 0.05 (denoted as * *p* < 0.05, *p* < 0.01, *** *p* < 0.001, and **** *p* < 0.0001; ns = not significant).

## 5. Conclusions

This study demonstrates that 6PPD and 6PPD-Q exert potent neurotoxic effects closely associated with AD and PD pathogenesis. Through integrated network toxicology, molecular docking, transcriptomic validation, and experimental approaches, we showed that both compounds cross the blood–brain barrier and trigger oxidative stress, neuroinflammation, mitochondrial dysfunction, and apoptosis-related signaling in the brain. 6PPD primarily impairs axonal integrity and oxidative-lipid homeostasis, whereas 6PPD-Q more aggressively promotes proteinopathy and microglial activation via GSK3B and TLR/MAPK pathways. CYCS and MAP2K1 serve as critical shared nodes. These results provide mechanistic evidence linking tire wear pollutants to neurodegenerative risk and underscore the urgent need for safer alternatives to 6PPD. Future chronic exposure studies and human-relevant models are warranted to validate these findings.

## Figures and Tables

**Table 1:** Predicted probabilities of organ toxicity for 6PPD and 6PPD-Q.

Chemical	Classification	Target	Prediction	Probability
6PPD	Organ toxicity	Hepatotoxicity	Inactive	0.73
		Neurotoxicity	Active	0.66
		Nephrotoxicity	Inactive	0.89
		Respiratory toxicity	Active	0.58
		Cardiotoxicity	Inactive	0.87
6PPD-Q	Organ toxicity	Hepatotoxicity	Inactive	0.65
		Neurotoxicity	Active	0.57
		Nephrotoxicity	Inactive	0.70
		Respiratory toxicity	Inactive	0.56
		Cardiotoxicity	Inactive	0.77

**Table 2:** Predicted probabilities of toxicity end points for 6PPD and 6PPD-Q.

Chemical	Classification	Target	Prediction	Probability
6PPD	Toxicity end points	Carcinogenicity	Inactive	0.59
		Immunotoxicity	Inactive	0.97
		Mutagenicity	Inactive	0.86
		Cytotoxicity	Inactive	0.75
		BBB-barrier	Active	0.84
		Ecotoxicity	Active	0.81
		Clinical toxicity	Inactive	0.55
		Nutritional toxicity	Inactive	0.71
6PPD-Q	Toxicity end points	Carcinogenicity	Inactive	0.56
		Immunotoxicity	Inactive	0.67
		Mutagenicity	Inactive	0.71
		Cytotoxicity	Inactive	0.70
		BBB-barrier	Active	0.65
		Ecotoxicity	Inactive	0.52
		Clinical toxicity	Active	0.53
		Nutritional toxicity	Inactive	0.64

**Table 3:** Top 3 terms of MCODE analysis for 6PPD targets.

Disease	MCODE	GO	Description	Log10(*p*)
AD	MCODE_1	R-HSA-193692	Regulated proteolysis of p75NTR	−23.7
		WP5124	Alzheimer 39 s disease	−18.4
		WP2059	Alzheimer 39 s disease and miRNA effects	−18.3
	MCODE_2	GO:0070098	chemokine-mediated signaling pathway	−13.8
		GO:1990868	response to chemokine	−13.5
		GO:1990869	cellular response to chemokine	−13.5
	MCODE_3	GO:0030574	collagen catabolic process	−10.7
		GO:0010594	regulation of endothelial cell migration	−10.5
		R-HSA-1679131	Trafficking and processing of endosomal TLR	−10.3
	MCODE_4	R-HSA-9027307	Biosynthesis of maresin-like SPMs	−9.6
		GO:0016098	monoterpenoid metabolic process	−9.6
		R-HSA-9018682	Biosynthesis of maresins	−9.2
	MCODE_5	GO:1904862	inhibitory synapse assembly	−15.5
		WP4159	GABA receptor signaling	−15.1
		GO:0007214	gamma-aminobutyric acid signaling pathway	−15.1
	MCODE_6	R-HSA-418555	G alpha (s) signalling events	−9.2
		GO:0007189	adenylate cyclase-activating G protein-coupled receptor signaling pathway	−8.9
		GO:0007188	adenylate cyclase-modulating G protein-coupled receptor signaling pathway	−8.2
	MCODE_7	GO:1903047	mitotic cell cycle process	−5.3
		GO:0000278	mitotic cell cycle	−5.1
		R-HSA-1640170	Cell Cycle	−4.9
PD	MCODE_1	R-HSA-193692	Regulated proteolysis of p75NTR	−16.6
		M153	PID P75 NTR PATHWAY	−15.5
		GO:0016098	monoterpenoid metabolic process	−8.9
	MCODE_2	R-HSA-9027307	Biosynthesis of maresin-like SPMs	−8.9
		GO:0010038	response to metal ion	−8.5
	MCODE_3	GO:0071396	cellular response to lipid	−18.8
		WP2374	Oncostatin M signaling	−16.8
		WP2034	Leptin signaling	−16.0
	MCODE_4	WP5324	Octadecanoid formation from linoleic acid	−13.7
		R-HSA-2142753	Arachidonate metabolism	−10.9
		GO:0019369	arachidonate metabolic process	−10.8
	MCODE_5	GO:0010506	regulation of autophagy	−7.6
		GO:0062013	positive regulation of small-molecule metabolic process	−6.2
		GO:2001242	regulation of intrinsic apoptotic signaling pathway	−6.0

**Table 4:** Top 3 terms of MCODE analysis for 6PPD-Q targets.

Disease	MCODE	GO	Description	Log10(*p*)
AD	MCODE_1	R-HSA-1679131	Trafficking and processing of endosomal TLR	−12.7
		GO:0043410	positive regulation of MAPK cascade	−12.0
		GO:0010817	regulation of hormone levels	−11.3
	MCODE_2	hsa04914	Progesterone-mediated oocyte maturation	−10.5
		WP2374	Oncostatin M signaling	−9.1
		WP5158	Urotensin II-mediated signaling	−8.8
	MCODE_3	R-HSA-9017802	Noncanonical activation of NOTCH3	−21.7
		GO:0034205	amyloid-beta formation	−21.3
		GO:0007220	Notch receptor processing	−21.3
	MCODE_4	CORUM:5877	MAP2K1-BRAF-RAF1-YWHAE-KSR1 complex	−10.7
		R-HSA-5674499	Negative feedback regulation of MAPK pathway	−10.4
		hsa05220	Chronic myeloid leukemia	−9.7
	MCODE_5	GO:0007169	cell surface receptor protein tyrosine kinase signaling pathway	−7.4
		GO:0007167	enzyme-linked receptor protein signaling pathway	−6.7
		GO:0030098	lymphocyte differentiation	−5.4
PD	MCODE_1	hsa04723	Retrograde endocannabinoid signaling	−9.2
		GO:0006468	protein phosphorylation	−7.5
		GO:0098657	import into cell	−7.5
	MCODE_2	R-HSA-9017802	Noncanonical activation of NOTCH3	−17.8
		GO:0007220	Notch receptor processing	−17.4
		GO:0034205	amyloid-beta formation	−17.4
	MCODE_3	M101	PID HDAC CLASSI PATHWAY	−9.5
		M115	PID REG GR PATHWAY	−9.1
		hsa05165	Human papillomavirus infection	−9.0
	MCODE_4	R-HSA-418555	G alpha (s) signalling events	−11.5
		GO:0007189	adenylate cyclase-activating G protein-coupled receptor signaling pathway	−11.2
		GO:0007188	adenylate cyclase-modulating G protein-coupled receptor signaling pathway	−10.2
	MCODE_5	R-HSA-1679131	Trafficking and processing of endosomal TLR	−17.2
		R-HSA-168898	Toll-like Receptor Cascades	−11.3
		GO:0030574	collagen catabolic process	−10.8
	MCODE_6	R-HSA-9658195	Leishmania infection	−8.4
		R-HSA-9824443	Parasitic Infection Pathways	−8.4
		hsa05130	Pathogenic Escherichia coli infection	−8.0
	MCODE_7	WP5475	Hallmark of cancer sustaining proliferative signaling	−10.0
		GO:0051897	positive regulation of phosphatidylinositol 3-kinase/protein kinase B signal transduction	−8.6
		GO:0001934	positive regulation of protein phosphorylation	−8.5

## Data Availability

The data presented in this study are available upon request from the corresponding author. The transcriptomic datasets analyzed during the current study are available in the GEO repository, under accession numbers GSE8397 and GSE5281.

## Supplementary Materials

The following supporting information can be downloaded at https://www.mdpi.com/article/10.3390/toxics14060504/s1. Table S1: Compilation of targets of 6PPD and 6PPD-Q; Table S2: Sources and analysis of GEO datasets related to neurodegenerative diseases; Table S3: Primer sequences used for RT-qPCR.

## Abbreviations

The following abbreviations are used in this manuscript:

6PPD	N-(1,3-dimethylbutyl)-N′-phenyl-p-phenylenediamine
6PPD-Q	6PPD-quinone
PPDs	p-phenylenediamine antioxidants
TRWPs	Tire and road wear particles
AD	Alzheimer’s disease
PD	Parkinson’s disease
CNS	Central nervous system
BBB	Blood–brain barrier
CSF	Cerebrospinal fluid
Aβ	Amyloid-β
ROS	Reactive oxygen species
PPI	Protein–protein interaction
GO	Gene Ontology
KEGG	Kyoto Encyclopedia of Genes and Genomes
MCC	Maximal clique centrality
SMILES	Simplified molecular-input line-entry system
GEO	Gene Expression Omnibus
MCODE	Molecular Complex Detection
RT-qPCR	Reverse transcription quantitative polymerase chain reaction
BV2	Murine microglial cell line BV2
MAP2K1	Mitogen-activated protein kinase kinase 1
MAPK8	Mitogen-activated protein kinase 8
EGFR	Epidermal growth factor receptor
GSK3B	Glycogen synthase kinase 3 beta
CYCS	Cytochrome c, somatic
BRAF	B-Raf proto-oncogene, serine/threonine kinase
TNF-α	Tumor necrosis factor alpha
IL-1β	Interleukin-1 beta
IL-6	Interleukin-6
IFN-γ	Interferon gamma
