# Supplementary material for: Molecular Mechanisms of 6PPD and 6PPD-Q Toxicity in Neurodegenerative Diseases: A Network Toxicology and Experimental Validation Study
Source: Toxics. 2026 Jun 10;14(6):504. doi: 10.3390/toxics14060504 (PMC13308044; doi:10.3390/toxics14060504)
Supplement: Supplementary file 1 [file toxics-14-00504-s001.zip › toxics-4335862-supplementary.pdf]

Table S1. Compilation of targets of 6PPD and 6PPD-Q

**Compound**	**Platform**	**Representative query examples**	**Species filter**
6PPD	SwissTargetPrediction	PubChem-derived canonical SMILES of 6PPD	*Homo sapiens*
6PPD	TargetNet	PubChem-derived canonical SMILES of 6PPD	*Homo sapiens*
6PPD	ChEMBL	"6PPD"; "N-(1,3-dimethylbutyl)-N′-phenyl-p-phenylenediamine"; PubChem-derived canonical SMILES	*Homo sapiens*
6PPD	STITCH	"6PPD"; available chemical identifier; PubChem-derived canonical SMILES	*Homo sapiens*
6PPD-Q	SwissTargetPrediction	PubChem-derived canonical SMILES of 6PPD-Q	*Homo sapiens*
6PPD-Q	TargetNet	PubChem-derived canonical SMILES of 6PPD-Q	*Homo sapiens*
6PPD-Q	ChEMBL	"6PPD-Q"; "6PPD-quinone"; PubChem-derived canonical SMILES	*Homo sapiens*
6PPD-Q	STITCH	"6PPD-Q"; "6PPD-quinone"; available chemical identifier; PubChem-derived canonical SMILES	*Homo sapiens*

Table S2. Sources and analysis of GEO datasets related to neurodegenerative diseases.

**Disease**	**Dataset**	**Experimental Design Parameters**	**Source of Biological Samples**
Alzheimer's disease	GSE5281	This research involved the collection of brain samples from 150 donors at three Alzheimer's Disease Centers (Arizona, Duke, and Washington University). Six AD- and aging-related regions were targeted for sampling. Following cell lysis and total RNA extraction, the samples underwent a double-round amplification process. Finally, gene expression patterns were analyzed using the Affymetrix U133 Plus 2.0 array.	*Homo sapiens*, hippocampus
Parkinson's disease	GSE8397	Post mortem brain tissue samples from Parkinson's disease and control cases (substantia nigra, split into medial and lateral portions, and frontal cortex) were prepared for this study. In total, 47 individual tissue samples were analyzed using one A and one B GeneChip per sample, i.e. 15 samples of medial parkinsonian SN, 9 samples of lateral parkinsonian SN, 8 medial nigra control samples and 7 lateral nigra control samples. Lateral and medial nigra samples were from the same cases.	*Homo sapiens*, substantia nigra

Table S2. Primer sequences used for RT-qPCR

Gene	Forward Primer (5'→3')	Reverse Primer (5'→3')
β-actin	CATTGCTGACAGGATGCAGA	GATGGTGGGATGGGTCAGAA
Cycs	GGTGCTGGAGACGGTAATCT	CGTGGCCTTTCCACATTCTC
Map2k1	CCGAGGACAGTAACGTCCTG	CTCTGGTTAGCCACAGCACT
Mapk8	CATCACATCCACATGCGTGA	CTGGCAGGGGTAATCTGTCC
Egfr	TGCTGGATCATCCTCTTCCT	TGGAGCCAGACTTCTTCCAT
Hif1a	GATGATGAGCGATGCGAGTC	GCACACCAGTGAGGTCTTTG
BraF	AGTTGGCGAGTTCCCAAGAT	GCCCATCCGAGATAAACTCCC
Gsk3b	CCAACTGCAAGCTAGGTGTG	GGCAGCAGTTGATTCGATCC
Tnf	GGTGCCTATGTCTCAGCCTCTT	GCCATAGAACTGATGAGAGGGAG
Il1b	TGGACCTTCCAGGATGAGGACA	GTTCATCTCGGAGCCTGTAGTG
Il6	CCACTTCACAAGTCGGAGGCTTA	GCAAGTGCATCATCGTTGTTCATAC
Ifng	TTACTACCTTCTTCAGCAACAGCAA	CTGGTGGACCACTCGGATGAG
